# Implementation and validation of the WHO ICOPE framework in andorra: a nationwide pilot study

**DOI:** 10.1016/j.jarlif.2025.100033

**Published:** 2025-12-31

**Authors:** Eva Heras, Jan Missé, Encarnació Ulloa, Gemma Ballester, Maria Anglada, Oliver Valero

**Affiliations:** a Department of Ageing and Health, Andorran Health Service (SAAS), Escaldes-Engordany, Andorra; b Primary Care Nursing, Andorran Health Service (SAAS), Encamp, Andorra; c Long-Term Care Center, Andorran Health Service (SAAS), Andorra la Vella, Andorra; d Department of Mathematics, Universitat Autònoma de Barcelona, Barcelona, Spain

**Keywords:** Healthy ageing, Intrinsic capacity, ICOPE, Community-based intervention

## Abstract

**Background:**

Healthy ageing relies on preserving intrinsic capacity (IC), the combination of an individual’s physical and mental capacities. The World Health Organization (WHO) developed the Integrated Care for Older People (ICOPE) framework as a person-centred care pathway to identify declines in IC and link older adults to appropriate interventions. The ICOPE model outlines a four-step approach involving basic community-based assessment, in-depth clinical evaluation, personalized care planning, and regular monitoring. However, evidence on large-scale implementation of this framework remains limited.

**Objective:**

To assess the feasibility and diagnostic accuracy of Step 1 screening across all seven municipalities of Andorra, and to link individuals to tailored interventions.

**Methods:**

From 2020 to 2025, 874 community-dwelling adults ≥ 60 years underwent Step 1 screening followed by full Step 2 assessment, using instruments from the WHO ICOPE Handbook.

Local adaptations included a clock-drawing test, the Montreal Cognitive Assessment (MoCA), Body Mass Index (BMI), and frequency-based scoring of the Patient Health Questionnaire-9 (PHQ-9). Diagnostic metrics (sensitivity, specificity, and Cohen’s kappa [κ]) were calculated for each domain. Referrals and interventions were tracked.

**Results:**

Of 857 participants analysed, Step 2 showed the highest prevalence of impairment in hearing (55 %) and cognition (39 %). Step 1 sensitivity improved notably after adaptations: cognition (+12 percentage points), mood (+30 percentage points), and nutrition (+6 percentage points), with vision specificity rising from 17 % to 99.5 %. Overall, 1 182 referrals were generated; 8 % joined a 12-week multicomponent programme including physical activity, nutrition and psychosocial support.

**Conclusion:**

The ICOPE model is feasible at national scale. Local adaptations significantly enhanced screening accuracy without added burden. The Andorran experience offers a scalable and replicable model for other countries or regions and highlights the value of embedding healthy ageing in community care.

## Highlights

• Nationwide implementation of the WHO ICOPE framework in Andorra.
• Step 1 screening validated against full Step 2 assessments in 857 older adults.
• Local adaptations improved sensitivity and specificity across Intrinsic Capacity domains.
• 60 % of screen-positives referred to community-based programmes.
• Andorra offers a scalable model for integrated healthy ageing strategies.

## 1 Introduction

Healthy ageing is defined by the World Health Organization (WHO) as “the process of developing and maintaining the functional ability that enables well-being in older age.” [1] Functional ability derives from a person’s intrinsic capacity (IC)**,** the sum of physical and mental abilities, and the environments in which they live [2]. Safeguarding IC is a cornerstone of the UN Decade of Healthy Ageing 2021–2030 [3].

The ICOPE approach is structured as a four-step pathway implemented primarily through primary care services [4]. It begins with a basic assessment and initial intervention (Step 1), followed by in-depth clinical assessment (Step 2), development of a personalized care plan (Step 3), and regular monitoring and follow-up (Step 4). This model enables integrated, multidisciplinary care aligned with older adults’ values, priorities, and social context, and aims to delay or prevent dependency and institutionalization.

Pilot studies from France, China, Singapore and Spain, demonstrate that Step 1 is feasible and highly sensitive in primary-care settings [[5], [6], [7], [8], [9]].

Preliminary data from Andorra’s first municipal rounds, Canillo (2020) and Escaldes-Engordany (2024), were showcased in the WHO Clinical Consortium on Healthy Ageing meeting reports, positioning the Andorran experience within the global learning network [10,11].

Andorra is a European micro-state with a resident population of 87,971 persons as of June 30, 2025, served by a single public healthcare provider, the Andorran Health Service (SAAS). According to official national statistics, 19,448 residents (22.1 %) were aged 60 years or older [12], highlighting the importance of community-level strategies to promote healthy ageing.

Between 2020 and 2025, the Andorran Health Service scaled the ICOPE programme to all seven municipalities**.**

The present study reports on that roll-out, with three objectives:

– To assess the feasibility and scalability of nationwide implementation of ICOPE;
– To validate Step 1 screening by comparing it with Step 2 applied to every participant;
– To link individuals with confirmed impairments to appropriate interventions, in alignment with ICOPE Steps 3 and 4.

## 2 Methods

### 2.1 Study design and setting

This prospective, community-based implementation study was conducted from July 2020 to March 2025. The intervention was deployed sequentially across all seven municipalities, coordinated by the SAAS in partnership with local governments.

These municipalities vary in both demographic and geographic characteristics. Andorra la Vella and Escaldes-Engordany are the most urbanised and densely populated municipalities, hosting the largest healthcare and administrative infrastructures. In contrast, Canillo and Ordino are predominantly rural, with smaller and more dispersed populations. Although both have access to nearby primary care centres and are within short driving distance of the hospital, their geographic and demographic features differ from more urbanised areas, which may influence how health programmes are delivered and perceived. The remaining municipalities, Encamp, La Massana, and Sant Julià de Lòria present mixed profiles, combining small urban centres with surrounding rural areas. This territorial diversity enabled the assessment of ICOPE feasibility across different settings, thereby enhancing the external validity of the findings.

To support implementation, the SAAS signed formal cooperation agreements with each of the seven municipal councils. These agreements, co-signed by the municipal mayor and the general manager of the SAAS, established that, during a predefined period, free functional screenings would be offered to residents aged 60 and over who wished to participate. Municipalities committed to providing suitable venues for assessments and assisting with outreach and mobilisation efforts.

At the outset, a national coordination meeting was held with political leaders, health professionals, and social care representatives from all municipalities to present the programme and highlight the value of screening intrinsic capacity. This was followed by local planning meetings with municipal social and ageing-services leads, enabling the tailoring of the implementation to local structures and fostering ownership at the community level.

Each local rollout phase lasted approximately two to three months, during which free screenings were offered and widely promoted at the community level.

In line with the cooperation agreements, local feedback sessions were held at the end of each rollout phase to share descriptive findings with political stakeholders and participants. Accordingly, results are presented by municipality to reflect this structure.

### 2.2 Participants and assessments

A total of 874 community-dwelling adults aged ≥ 60 years were enrolled; 857 (98.1 %) completed both Step 1 and Step 2 and constitute the analytic sample.

Participants were proactively invited through coordinated outreach led by primary care centres and municipal social services, as part of formal agreements signed with each local government.

The recruitment strategy combined official press releases, social media campaigns, signed agreements between SAAS and each municipality, and proactive outreach in primary care centres. In addition, word-of-mouth referrals by previous participants played a substantial role in facilitating enrolment. Each municipality provided a multipurpose venue, typically located within communal buildings (e.g., cultural centres or public service halls). These spaces had to meet specific requirements, including a minimum of four meters of unobstructed floor length (to allow physical performance testing such as the SPPB), a quiet room suitable for hearing assessment, Wi-Fi access to enable use of tablet-based digital forms, and the possibility to connect a printer for providing participants with personalised reports.

Assessments were conducted face to face, lasting approximately 60 min, by trained primary care nurses in collaboration with professionals from the Department of Ageing and Health, including geriatricians and a physical exercise specialist.

This decentralised and participant-centred approach contributed to the high completion rate observed and ensured strong engagement and successful nationwide deployment of the ICOPE programme.

All assessments followed a standardized protocol delivered by trained staff who underwent one year of exposure to ICOPE tools and procedures, including practical workshops and supervised sessions. Nurses were responsible for cognitive, nutritional, emotional, sensory and continence domains, while physical performance assessments such as the SPPB were administered by the exercise specialists.

### 2.3 ICOPE screening and care pathway

The study followed the domains and questions specified in the WHO ICOPE Handbook [13] and the companion ICOPE Mobile App (beta version). These items were transferred to custom digital forms used on tablet computers (proprietary forms in 2020–2022; REDCap thereafter [14]). Every participant completed Step 1 and, irrespective of the result, Step 2, thereby eliminating verification bias. Step 2 served as the reference assessment, classifying participants as ‘impaired’ or ‘non-‘impaired’ in each intrinsic-capacity domain.

In addition to the standard Step 1 and Step 2 items specified in the WHO ICOPE Handbook and App, selected domains were locally adapted based on clinician feedback and field experience, to enhance feasibility and diagnostic accuracy. These adaptations, implemented in parallel with standards items, included:

• Cognition: All participants completed the MoCA and Clock Drawing Test, in addition to standard Step 2 cognitive testing.
• Nutrition: The BMI was calculated systematically after completing Step 1 nutritional screening.
• Vision: The Step 1 question was refined to differentiate between corrected and uncorrected visual issues.
• Mood: In addition to completing the PHQ-9 without scoring (as per WHO guidance), participants were asked to indicate the frequency of each symptom, as in the original PHQ-9, allowing deeper analysis.

These adaptations did not replace the original ICOPE items but were conducted in parallel. As such, all participants were assessed using both the standard tools and the adapted items. According to staff feedback, the adaptations did not result in any perceived increase in time or workload, although no objective timing data were collected.

Table 1 summarises the instruments used for both steps and highlights the local adaptations introduced during implementation.

**Table 1:** Step 1 and Step 2 instruments and local adaptations.

Domain	Step 1 items (ICOPE)	Step 2 reference (app default)	Local adaptations
Cognition	3-word recall + orientation	GPCOG[18]	+ Clock-drawing[19] in Step 1; + MoCA[20] alongside GPCOG in Step 2
Mobility	5 chair-rises ≤ 14 s	SPPB[21]	—
Nutrition / Vitality	Weight loss + ↓ appetite	MNA-SF[22]	+ BMI item in Step 1
Vision	Self-reported visual problems	Snellen chart	Step 1 wording changed to “uncorrected” visual problems
Hearing	Whisper test (0.6 m)[23]	Pure-tone audiometry	—
Mood	2 PHQ-9 items (yes/no)	PHQ-9 (non-frequency) [24]	Step 2 replaced by full, frequency-anchored PHQ-9

**Note:** Step 1 items were drawn from the WHO ICOPE Mobile App (clinician version). Local adaptations were introduced progressively in response to feasibility feedback from field implementation.

Participants identified as impaired in one or more domains were referred to appropriate services via six main pathways:

1. General practitioner and, when indicated, ear, nose and throat specialists (ENT) or ophthalmologists.
2. Community exercise facilities / municipal gyms offering frailty-adapted programmes.
3. A Multi-Domain Group-Based Intervention (AMICOPE) [16]. AMICOPE is a structured 12-week multicomponent programme originally developed in Catalonia to enhance physical activity, nutrition and psychosocial well-being in older adults with early declines in intrinsic capacity. It was adapted locally for group-based delivery in municipal facilities [15].
4. Municipal workshops (memory-training sessions, adapted dance, Qigong, aqua gym, level-appropriate exercise classes, guided hikes, cultural outings and other community activities).
5. Community social-work services.
6. Primary-care nursing follow-up.

All community-based exercise programmes, including AMICOPE and municipal workshops, were offered free of charge. While no formal medical prescription was required, participation was generally recommended by the nurse conducting the Step 2 assessment, based on the individual’s profile and specific functional needs.

This referral pathway differs from routine care in that it was nurse-led, rather than initiated by the patient or their general practitioner. Trained nurses, based on the ICOPE results and in coordination with geriatricians and physical activity professionals, were empowered to recommend direct referrals not only to GPs but also to ENT or ophthalmology specialists when specific deficits were identified. This model, made possible through the framework of the ICOPE pilot, enabled timely, tailored interventions at the community level and represents a departure from standard referral flows, which typically rely on patient-initiated contact with primary care.

Immediately after assessment, participant received:

– A printed report summarising their results.
– A Vivifrail exercise booklet[15] tailored to their physical level.
– Cognitive-stimulation worksheets tailored to MoCA score.

These tools supported the creation of personalised care plans. Although only 12 % of participants were referred to general practitioners, this reflected the early-prevention profile of the population, which was composed mostly of independent, community-dwelling older adults without overt clinical conditions. Most impairments identified related to sensory or functional decline rather than acute medical needs, which explains the higher number of referrals to specialists (e.g. 260 to ENT, 37 to ophthalmologists) and community services.

Coordination across nurses, social workers, fitness specialists and municipal services ensured a multidisciplinary approach beyond the traditional medical model.

Though implemented within a research framework, this model has provided valuable insight for the potential formalisation of a nurse-led, community-integrated care pathway in line with WHO ICOPE principles.

### 2.4 Data handling and statistical analysis

Data were captured in proprietary forms and REDCap and analysed in SAS 9.4. For each domain we calculated sensitivity, specificity, overall accuracy and Cohen’s κ (95 % confidence intervals) by comparing Step 1 with Step 2. These indices were re-estimated before and after local adaptations (clock-drawing, BMI item, revised vision question) to gauge their impact. Missing data were < 1 % per variable and handled by complete-case analysis.

### 2.5 Ethics

The study complied with the principles of the Declaration of Helsinki (2013) and was approved by the Andorran Health Service Research Ethics Committee. Written informed consent was obtained from all participants.

## 3 Results

### 3.1 Participant flow and baseline characteristics

**Fig. 1:**
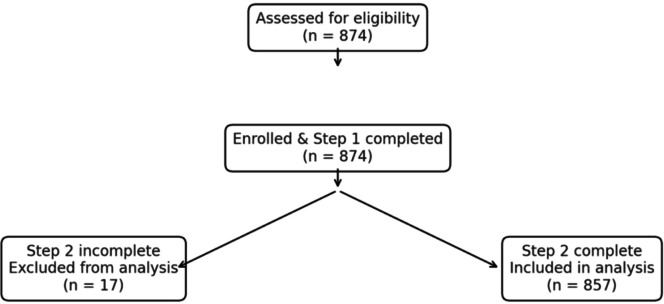
CONSORT flow diagram of participant inclusion.

Between July 2020 and March 2025, 874 community-dwelling adults ≥ 60 y were enrolled (Fig. 1). After excluding 17 individuals with incomplete Step 2 data, 857 participants (98.1 %) formed the analytic sample. Mean age was 76 ± 6.5 y and 31 % were men; baseline characteristics were similar across the seven municipalities.

Step 1 positivity rates varied by region and domain (Table 2).

**Table 2:** Participant characteristics and Step 1 screen-positive rates by municipality.

Variable	Canillo	Encamp	Ordino	La Massana	Andorra la Vella	Escaldes-Engordany	Sant Julià de Lòria	**Total**
**Number of participants**	99	88	42	91	148	269	137	**874**
**Mean age (sd)**	73.8 (6.5)	76.5 (7.3)	75.6 (7.1)	76.3 (6.5)	76.8 (7.5)	76.4 (6.4)	76.7 (5.1)	**76.3 (6.5)**
**Men %**	48.8	18.2	26.8	26.1	23.6	33.1	38.7	**31.4**
**% Step 1 screen-positive**								
• Cognition %	46.8	77.3	57.1	63.7	57.8	58.4	38.0	**56.2**
• Mobility %	18.2	26.1	23.8	27.5	27.7	17.5	12.4	**20.7**
• Nutrition %	12.1	0.0	4.9	2.2	4.9	14.5	19.7	**10.3**
• Vision %	83.8	94.3	90.2	82.2	91.8	72.5	N/A	**82.7**
• Hearing %	31.3	44.3	70.7	55.1	33.3	36.1	22.6	**41.1**
• Mood %	33.3	18.2	14.6	9.9	24.3	17.1	44.5	**23.7**

**Note:** Screen-positive” refers to participants who, during the baseline Step 1 screening (before local adaptations), were identified as potentially impaired in each respective domain of intrinsic capacity. N/*A* = data not available.

Cognitive risk ranged from 38.0 % in Sant Julià de Lòria to 77.3 % in Encamp. These differences likely reflect a combination of factors. The Step 1 cognitive screen is based on orientation and short-term memory questions, which are sensitive but not specific. In Encamp, although 77.3 % screened positive for cognitive risk in Step 1, only 41 % met Step 2 criteria, suggesting high false-positive rates. Moreover, Encamp was the last parish to be screened (2025), by which time local health and social workers may have referred individuals already known or suspected to have cognitive difficulties. By contrast, Sant Julià was among the first municipalities included (2021), with less targeted recruitment and less operational experience. These temporal and procedural differences may have influenced the composition of screened populations.

No statistical comparisons between municipalities were planned or conducted. Differences are presented descriptively to reflect the real-world, phased implementation and local context of each rollout.

Mobility risk was highest in La Massana (27.5 %) and Andorra la Vella (27.7 %) and lowest in Escaldes-Engordany (17.5 %) and Sant Julià (12.4 %). One plausible explanation is the promotion of physical activity directly by the municipalities. Both Escaldes and Sant Julià run regular group-based exercise programs for older adults, and several screened participants were active members of these initiatives, which may help preserve functional mobility.

Nutritional risk ranged from 0 % in Encamp to 19.7 % in Sant Julià. These variations may reflect socioeconomic differences or residual effects of the COVID-19 pandemic on appetite, isolation, or food access, especially in early screened regions.

Vision difficulties were flagged at high rates across all municipalities in Step 1 (range: 72.5–94.3 %). However, these rates reflected self-reported impairments, many of which were already corrected with glasses or lenses. To improve the screen’s specificity, we adapted the Step 1 item to explicitly ask whether the participant had any uncorrected visual difficulties. This adaptation helped distinguish intrinsic-capacity loss from corrected visual problems. In Step 2, confirmed visual impairment was present in only 5 % of participants, indicating that most Step 1 reports were not true deficits, but issues effectively managed with optical aids. As such, vision results from Step 1 were not considered a reliable marker for inter-regional comparison.

Hearing risk showed the widest range (22.6–70.7 %), with the highest prevalence in Ordino (70.7 %). Participants from Ordino live in more isolated rural settings and may have had less access to audiological care or corrective devices.

Mood symptoms were most flagged in Canillo (33.3 %) and Sant Julià (44.5 %), both of which were among the first parishes screened during or shortly after the first COVID-19 waves. Emotional burden linked to the pandemic may explain these findings.

### 3.2 Prevalence of intrinsic-capacity impairments (Step 2)

Hearing loss (55 %) and cognitive impairment (39 %) were the most frequent declines, whereas uncorrected vision loss was rare (5 %), likely reflecting prevalent optical correction. These prevalences define the denominators for diagnostic-accuracy calculations.

Participants with suspected cognitive impairment or hearing loss based on Step 2 results were referred to their general practitioner or to relevant specialists (e.g., otorhinolaryngologists) for further evaluation and management. In addition, screening results and recommendations were documented in everyone’s digital health record, facilitating continuity of care and enabling longitudinal follow-up by primary care teams.

Table 3 summarises how many participants met the predefined Step 2 cut-offs in each intrinsic-capacity domain.

**Table 3:** Prevalence of intrinsic-capacity impairments (Step 2 reference) **(*n* = 857)**.

Domain	Instrument & cut-off	Prevalence %
Cognition	MoCA < 26	39.2
Mobility	SPPB < 10	23.0
Nutrition	MNA-SF < 12	10.1
Vision	Snellen worse than 6/12	4.9
Hearing	Pure-tone average > 35 dB HL	55.2
Mood	PHQ-9 > 4	12.3

### 3.3 Diagnostic accuracy of the step 1 screen

Table 4 compares sensitivity, specificity and Cohen’s kappa (κ) for the original Step 1 items versus the locally adapted screen. Adding a clock-drawing test and using MoCA as a reference tool in Step 2 improved cognitive sensitivity by 12 percentage points (pp). *Re*-wording the vision question increased specificity from 17 % to 99.5 % (κ 0.07 → 0.60). Incorporating BMI raised nutritional sensitivity by 6 pp, and using the full frequency-anchored PHQ-9 boosted mood sensitivity by 30 pp while retaining acceptable specificity. Mobility and hearing metrics were unaltered, as no adaptations were introduced. Post-adaptation overall accuracy ranged 58 % - 90 %, highest for nutrition and mobility (Table 5).

**Table 4·:** Diagnostic accuracy of Step 1 before and after local adaptations.

Domain		Baseline Step 1			Adapted Step 1	
	Sens %	Spec %	κ	Sens %	Spec %	κ
Cognition	68.9	58.6	0.28	80.9	52.4	0.30
Mobility	65	93	0.60	—	—	—
Nutrition	48.9	94.1	0.43	54.7	93.7	0.46
Vision	83.3	17.2	0.001	50.0	99.5	0.60
Hearing	50.1	67.0	0.17	—	—	—
Mood	41.4	91.9	0.31	70.5	84.6	0.41

**Note***: Dashes* denote domains without local adaptation.

**Table 5:** **·**Destinations of referral orders and programme uptake (*n* = 857).

Destination / programme	n	% of participants
General practitioner (primary care)	101	12
**Ear, nose and throat/** Ophthalmology specialist	260/37	30/4
Municipal gyms / exercise facilities	150	18
AMICOPE 12-week multicomponent programme	68	8
Municipal workshops (memory, adapted dance, Qigong, aqua gym, graded exercise, guided hikes, cultural outings)	512	60
Social-work services	54	6

### 3.4 Destinations of referral orders

The most frequent clinical referral was to ear, nose and throat (260 participants, 30 %), followed by general practitioners (101 participants, 12 %).

At the community level, 150 individuals (18 %) were referred to municipal exercise facilities, 68 (8 %) joined the AMICOPE multicomponent programme, and 512 (60 %) were referred to municipal workshops offering activities such as memory training, adapted dance, Qigong, aquagym, and cultural outings.

Social-work services managed 54 participants (6 %) presenting with social isolation, financial hardship or complex support needs.

No assessment or intervention-related adverse events were reported.

It should be noted that no formal follow-up was conducted to confirm whether participants completed the referrals or interventions. As such, these figures reflect referral orders issued based on the ICOPE assessment, not verified programme adherence.

## 4 Discussion

### 4.1 Principal findings

This nation-wide pilot confirms that the WHO ICOPE pathway is feasible in a micro-state context and that a brief, locally adapted Step 1 screen can detect early IC declines with acceptable accuracy. Feasibility was supported by several indicators: (1) high completion rate (98.1 % of enrolled participants completed both steps); (2) consistent implementation across all seven municipalities, each securing physical venues and outreach coordination through formal agreements; (3) successful training and participation of primary care nurses in screening delivery; and (4) no adverse events or major logistical setbacks during the five-year rollout. Although no structured survey was conducted among health workers, informal debriefings and regular team coordination meetings reported high acceptability and integration into local workflows. Scalability was demonstrated by the programme’s progressive extension from one pilot municipality to full national coverage between 2020 and 2025. Almost half of screened participants required a referral, demonstrating the pathway’s capacity to mobilise integrated health and social care resources at scale. Sensitivities were highest for cognition (81 %) and mood (71 %) after adaptation, while the vision question achieved near-perfect specificity (99.5 %).

### 4.2 Comparison with international ICOPE step 1 implementation experiences

The results of this nationwide pilot in Andorra are broadly consistent with previous ICOPE Step 1 implementation experiences across France, China, Singapore, and Spain [5–9]. In line with reports from large-scale digital cohorts in France and community-based studies in Asia, our pilot achieved a near-complete initial completion rate (98.1 %) and demonstrated high sensitivity in key domains, particularly cognition and mood.

For example, sensitivity in the cognition domain reached 81 % in Andorra, compared with 89 % in the Spanish VIMCI study and up to 100 % in a Chinese community-based cohort.

Specificity values in our study also followed known trends, with high specificity in vision (99.5 %) and lower performance in hearing, as reported in the literature.

The proportion of participants with abnormal Step 1 results was lower in our setting (∼50 %) than in most professional-led cohorts in France, where abnormal screening rates exceeded 90 %.

This discrepancy may be partly explained by contextual differences: our screening was conducted in community venues, targeting a broad population of older adults, including relatively healthy individuals, whereas many comparative studies focused on frailer or more clinically complex populations.

Furthermore, domain-specific performance varied across studies. As observed in other countries, vision, cognition, and hearing were consistently among the most frequently impaired domains. Variations in vitality and mobility detection were also noted across studies. In our setting, these domains were assessed using standardized ICOPE Step 1 items: the “Chair Rise Test” for mobility, and two straightforward vitality questions regarding recent unintentional weight loss and appetite. Given the simplicity and universality of these items, the reasons behind observed differences remain unclear and may require further investigation.

Overall, the Andorran experience reinforces existing evidence that Step 1 screening is feasible and informative across diverse contexts. It also highlights the importance of local adaptations, such as the inclusion of the clock drawing test and BMI measurement, which improved diagnostic yield. These results support the transferability of ICOPE across settings, while underlining the need for context-sensitive implementation strategies.

### 4.3 Strengths and limitations

**Strengths:** The presence of asingle public healthcare provider and pre-existing collaborations between the SAAS and the social affairs and older adult departments of each municipality, developed and strengthened through the EU-funded APTITUDE and APTITUDE PROXI projects [17], facilitated the rapid scale-up of the ICOPE pathway and its integration into both health and community services. These cross-border initiatives, conducted in partnership with institutions such as the Gérontopôle of Toulouse, the Public Hospital of Navarre, and the Health and Ageing Foundation of the Autonomous University of Barcelona, aimed to prevent dependency in adults aged 60 and over. Within this framework, Andorra implemented the ICOPE programme as part of a broader national strategy to promote healthy ageing and reduce the burden of frailty.

**Limitations**: Participation was voluntary, which may have led to an over-representation of healthier or more motivated individuals. Step 2 was applied to all participants, which could have inflated Cohen’s κ values compared to standard two-step procedures. Finally, the specificity of the hearing domain remained modest (67 %), likely due to the whisper test’s sensitivity to environmental noise.

### 4.4 Implications for practice and research

• **Policy:** The ICOPE framework can be embedded within existing primary care and community infrastructures to support early detection and integrated management of functional decline. Based on the results of this nationwide implementation, the Andorran Ministries of Health and Social Affairs are preparing to adopt ICOPE as a formal public health strategy. As of 2025, the ICOPE pathway continues to be implemented in clinical practice at the primary care level, led by nurses trained in geriatric assessment. The next step is to offer Step 1 screening to all adults aged 60 and over, either through self-assessment or with the support of primary care nurses. Results will be transmitted via the national electronic health record directly to the primary care nurse, who will be responsible for performing Step 2 if any Step 1 domain is impaired. Based on Step 2 outcomes, the nurse will activate a tailored intervention plan using a predefined map of resources and referral options. The integration of ICOPE into national policy aims to make healthy ageing a routine part of care, prevent dependency, and ensure timely, personalized responses to the needs of older adults.
• **Clinical practice:** The adapted Step 1 can be deployed by nurses in approximately 10 min. The added clock-drawing test and BMI item improve yield with negligible time cost. In Andorra, future implementation will be led by primary care nurses, who will receive Step 1 results, via digital self-assessment or assisted screening, and conduct Step 2 when indicated. Nurses will use predefined intervention pathways and community resource maps to support individualized care planning.
• **Research:** Future studies should evaluate cost-effectiveness, long-term effects on functional ability, and digital integration of Step 1 into electronic health records. Randomised implementation trials could also assess whether limiting Step 2 to screen-positives changes resource use or diagnostic validity.
• **Living-lab potential:** Owing to its unified health-care system, high digital readiness and strong municipal partnerships, Andorra offers a natural “living laboratory” for testing scalable healthy-ageing interventions and digital ICOPE tools, with transferability to both small countries and regional units in larger health systems. While the implementation occurred in a small-country setting, the lessons learned, and the tools adapted may inform regional or national strategies in larger health systems.

Beyond tool adaptation, this experience highlighted the high motivation of older adults to engage in preventive care and their openness to understanding and addressing early declines in intrinsic capacity. The referral process also helped reveal and connect individuals to existing community-based resources, such as exercise programmes and cultural workshops, that were previously underutilized or unknown. Primary care nurses emerged as trusted, community-embedded professionals ideally positioned to lead ICOPE deployment. Moreover, the programme demonstrated how health and social services can be effectively integrated, since many recommended actions were social in nature. These insights reinforce the value of ICOPE as both a clinical and community-building tool for anticipatory ageing care.

• Sustainability and evaluation considerations:

Although the pilot reached all seven municipalities, it covered less than 5 % of the older adult population. To ensure long-term sustainability and scale-up, the ICOPE model in Andorra must be consolidated through structural actions. These include the systematic inclusion of Step 1 screening in annual check-ups, the allocation of dedicated nurse coordinators within primary care centres, and the establishment of ongoing training programmes.

In parallel, robust monitoring and evaluation mechanisms should be put in place, including performance indicators (e.g., referral rates, follow-up adherence, functional outcomes), regular audits, and feedback loops involving both municipal and central health authorities.

The integration of these elements will not only guarantee continuity and quality but also help adapt the model over time to changing population needs and healthcare resources. These efforts could be progressively embedded within existing primary care frameworks, with institutional support from the national health system.

## 5 Conclusion

The Andorran pilot demonstrates that the WHO ICOPE framework can be implemented at country scale, that targeted adaptations substantially enhance screening accuracy, and that positive findings can be translated into integrated, multi-sectoral interventions. These results support wider adoption of ICOPE in similar settings and provide a validated Step 1 template for other European micro-states. However, for full national scale-up in Andorra, several key components remain under development. First, a digital version of Step 1, allowing older adults to complete the screening independently or with assistance, is being finalised and will need to be integrated with the national electronic health record system. This will enable automatic transmission of results to the primary care nurse, who will be responsible for completing Step 2 when needed. Second, structured training and workflow integration are required to empower primary care nurses to lead the ICOPE pathway, including decision-making and care coordination. Third, the creation of a comprehensive national map of available community resources is ongoing, to ensure that referral pathways and intervention programmes can be tailored to each municipality. Finally, a formal implementation strategy, led jointly by the Ministries of Health and Social Affairs, is needed to ensure national coordination, sustainable funding, and long-term monitoring of outcomes.

## Funding

The study was supported by the Andorran Health Service and, during its final year, received partial funding from the Government of Andorra through the supplementary call to POCTEFA 2023 (Ref. AUEP014-AND/2023).

## CRediT authorship contribution statement

**Eva Heras:** Writing – review & editing, Writing – original draft, Visualization, Validation, Supervision, Resources, Project administration, Methodology, Investigation, Funding acquisition, Data curation, Conceptualization. **Jan Missé:** Investigation, Methodology, Project administration. **Encarnació Ulloa:** Investigation, Methodology, Project administration, Resources. **Gemma Ballester:** Project administration, Resources, Supervision. **Maria Anglada:** Methodology, Resources. **Oliver Valero:** Formal analysis, Investigation, Methodology, Software.

## Declaration of competing interest

The authors declare the following financial interests/personal relationships which may be considered as potential competing interests:

EVA HERAS MUXELLA reports financial support was provided by Government of Andorra. If there are other authors, they declare that they have no known competing financial interests or personal relationships that could have appeared to influence the work reported in this paper.

## References

[1] World Health Organization (2015).

[2] Beard J.R., Si Y., Liu Z. (2021). Intrinsic capacity: validation of a new WHO concept for healthy ageing in a longitudinal Chinese study. J Gerontol A Biol Sci Med Sci.

[3] World Health Organization (2021).

[4] World Health Organization (2024). https://iris.who.int/handle/10665/380175.

[5] Tavassoli N., de Souto, Barreto P., Berbon C., Mathieu C., de Kerimel J., Lafont C. (2022 Jun). Implementation of the WHO integrated care for older people (ICOPE) programme in clinical practice: a prospective study. Lancet Healthy Longev.

[6] Berbon C., Takeda Catherine, Balardy L., Lafont Christine, Tavassoli N., Carrie Isabelle, Guyonnet S. (2024). Implementing the WHO ICOPE Program in Clinical practice: three years of lessons from monitoring 27,082 participants using the ICOPE monitor digital tool. J. Gerontol. A Biol. Sci. Med. Sci..

[7] Wang N., Liu X., Kong X., Sumi Y., Chhetri J.K., Hu L. (2024 Jan 2). Implementation and impact of the World Health Organization integrated care for older people (ICOPE) program in China: a randomised controlled trial. Age Ageing.

[8] Ma C.H.K., Chua D.Q.L., Tay L., Teo E.W.C., Ng W.C., Leung A.Y.M. (2024). The feasibility of implementing the WHO Integrated Care for Older People (ICOPE) framework in Singapore. J Frailty Aging.

[9] Luque Xavier Rojano i, Blancafort-Alias Sergi, Casanovas Susanna Prat, Forné Susanna, Martín Vergara. Pilar Fabregat Povill N., Royo Maria Vila (2023). Identification of decreased intrinsic capacity: performance of diagnostic measures of the ICOPE screening tool in community dwelling older people in the VIMCI study. BMC Geriatr.

[10] World Health Organization (2021). WHO Clinical Consortium on Healthy Ageing 2020: meeting report, 18–19 November 2020 (virtual).

[11] World Health Organization (2024). WHO Clinical Consortium on Healthy Ageing 2024: meeting report, 5–7 November 2024 (virtual).

[12] Departament d'Estadística del Govern d’Andorra. Estadística dels censos parroquials. Juny del 2025. Publicat el 17 de juliol de 2025. Disponible a: https://www.estadistica.ad, 2025.

[13] World Health Organization (2019).

[14] Harris P.A., Taylor R., Minor B.L. (2019). The REDCap consortium: building an international community of software platform partners. J Biomed Inform.

[15] Izquierdo M. (2019 Jul 1). Prescripción de ejercicio físico. El programa Vivifrail como modelo. Nutr Hosp.

[16] Blancafort Alias S., Cuevas-Lara C., Martínez-Velilla N., Zambom-Ferraresi F., Soto M.E., Tavassoli N. (2021 Jun 2). A multi-domain group-based intervention to promote physical activity, healthy nutrition, and psychological wellbeing in older people with losses in intrinsic capacity: AMICOPE development study. Int J Environ Res Public Health.

[17] APTITUDE PROXI – Promoting healthy ageing among cross-border rural populations. Interreg POCTEFA 2021–2027 [Internet] 2024. Available from: https://www.poctefa.eu/proyectos/efa018-01-id-aptitude-proxi/.

[18] Brodaty H., Kemp N.M., Low L.F. (2004). GPCOG: a screening test for cognitive impairment in older people. Int Psychogeriatr.

[19] Shulman K.I. (2000 Jun). Clock-drawing: is it the ideal cognitive screening test?. Int J Geriatr Psychiatry.

[20] Nasreddine Z.S., Phillips N.A., Bédirian V. (2005). The Montreal Cognitive Assessment (MoCA): a brief screening tool for mild cognitive impairment. J Am Geriatr Soc.

[21] Guralnik J.M., Simonsick E.M., Ferrucci L. (1994). A short physical performance battery assessing lower-extremity function. J Gerontol.

[22] Guigoz Y., Vellas B., Garry P.J. (1996). Assessing the nutritional status of the elderly: the Mini Nutritional Assessment as part of the geriatric evaluation. Nutr Rev.

[23] Eekhof J.A., de Bock G.H., de Laat J.A., Dap R., Schaapveld K., Springer M.P. (1996 Aug). The whispered voice: the best test for screening for hearing impairment in general practice?. Br J Gen Pract.

[24] Kroenke K., Spitzer R.L., Williams J.B. (2001). The PHQ-9: validity of a brief depression severity measure. J Gen Intern Med.

